# Supplementary material for: A machine learning based exploration of COVID-19 mortality risk
Source: PLoS One. 2021 Jul 2;16(7):e0252384. doi: 10.1371/journal.pone.0252384 (PMC8253432; doi:10.1371/journal.pone.0252384)
Supplement: S1 File — (PDF) [file pone.0252384.s001.pdf]

A machine learning based exploration of COVID-19 mortality risk

Mahdi Mahdavi[1,2¶], Hadi Choubdar[1,2¶], Erfan Zabeh[3¶], Michael Rieder[4,5,6,7], Safieddin Safavi-Naeini[8], Zsolt Jobbagy[9], Amirata Ghorbani[10], Atefeh Abedini[11], Arda Kiani[12], Vida Khanlarzadeh[6], Reza Lashgari[1*], and Ehsan Kamrani[4,8,13,14,15*]

[1]Institute of Medical Science and Technology (IMSAT), Shahid Beheshti University, Tehran, Iran
[2]Department of Medicine, Shahid Beheshti University of Medical Sciences, Tehran, Iran
[3]Department of Biomedical Engineering, Columbia University, United States
[4]Robarts Research Institute, Western Research Park, London, ON, Canada
[5]Department of Paediatrics, Children's Hospital of Western Ontario, London, ON, Canada
[6]Department of Medicine, Schulich School of Medicine and Dentistry, University of Western Ontario, London, ON, Canada
[7]CIHR-GSK Chair in Pediatric Clinical Pharmacology, Children's Health Research Institute, London, ON, Canada
[8]CIARS (Centre for Intelligent Antenna and Radio Systems), Department of ECE, University of Waterloo, Waterloo, Ontario, Canada
[9]Department of Pathology, Immunology and Molecular Pathology, Rutgers New Jersey Medical School, Newark, NJ, United States
[10]Department of Electrical Engineering, Stanford University, Stanford, CA, USA
[11]Chronic Respiratory Diseases Research Center, National Research Institute of Tuberculosis and Lung Diseases (NRITLD), Shahid Beheshti University of Medical Sciences, Tehran, Iran
[12]Tracheal Diseases Research Center, National Research Institute of Tuberculosis and Lung Diseases (NRITLD), Shahid Beheshti University of Medical Sciences, Tehran, Iran
[13]Department of Electrical and Computer Engineering, University of Waterloo, Waterloo, ON, Canada
[14]Wellman Center for Photomedicine, Harvard Medical School, Boston, MA, USA
[15]Harvard-MIT Health Science and Technology, Cambridge, MA, USA

[*]r_lashgari@sbu.ac.ir, ekamrani@uwaterloo.ca

[¶]These authors contributed equally to this work

S1 Table. List of machine learning input features and their definitions

Category	Feature	Definition
**Non-Invasive Features:**		
Demographic Features	1- Age	Age of the patient calculated from the patient's date of birth.
	2- Sex	Gender of the patient
Clinical Features	3- Blood Pressure max	The blood pressure of patients was assessed using the hospital's electronic BP monitors
	4- Blood Pressure min	The blood pressure of patients was assessed based on mmHg, using the hospital's electronic BP monitors
	5- Pulse Rate	The pulse rate of the patient. Measured using hospital pulse oximeter devices
	6- Respiratory Rate	Respiratory rate. Recorded by a nurse or physician as the count of patient respirations in one minute
	7- Temperature	The temperature of the patient; reported in ˚C and recorded using electronic forehead thermometers
	8- $SpO_{2,}$	The blood oxygen saturation of patients used in this study were measured in the room air (i.e., without oxygen support) using the hospital's pulse oximeters from patient fingers.
Comorbidities	9- Hypertension (HTN)	Presence of a history of hypertension. This information was extracted from the past medical history that was recorded from the patient by a hospital physician
	10- Diabetes Mellitus (DM)	Presence of a history of Diabetes Mellitus type I or II. This information was extracted from the past medical history that was recorded from the patient by a hospital physician
	11- Cardiovascular Disease	Presence of a history of cardiovascular disorders. History of Ischemic Heart Diseases (IHDs), Myocardial Infarctions (MI), or Heart Failure (HF) was considered as a positive history of cardiovascular disease. This information was extracted from the past medical history that was recorded from the patient by a hospital physician.
**Invasive Features:**		
Complete Blood Count (CBC)	12- WBC<br>13- Neutrophil*<br>14- Lymphocyte*<br>15- RBC<br>16- HB<br>17- HCR<br>18- Plt<br>19- MCV<br>20- MCH	Obtained from venous blood samples analyzed in the central laboratory of the hospital.

	21- MCHC<br>22- RDW<br>23- ESR	
Coagulation	24- PT<br>25- PTT<br>26- INR	Obtained from venous blood samples analyzed in the central laboratory of the hospital.
Biochemistry	27- BUN<br>28- Cr<br>29- AST<br>30- ALT<br>31- ALKp<br>32- LDH	Obtained from venous blood samples analyzed in the central laboratory of the hospital.
Blood Gas Test	33- PH<br>34- PCO2<br>35- PO2<br>36- HCO3<br>37- BE	Obtained from venous blood samples analyzed in the central laboratory of the hospital.

* Absolute Neutrophil and Lymphocyte Count: Number of neutrophils and lymphocytes in the blood sample of the patient. Calculated via multiplying the number of WBCs by neutrophil and lymphocyte ratio from CBC lab results

Flowchart of the study population

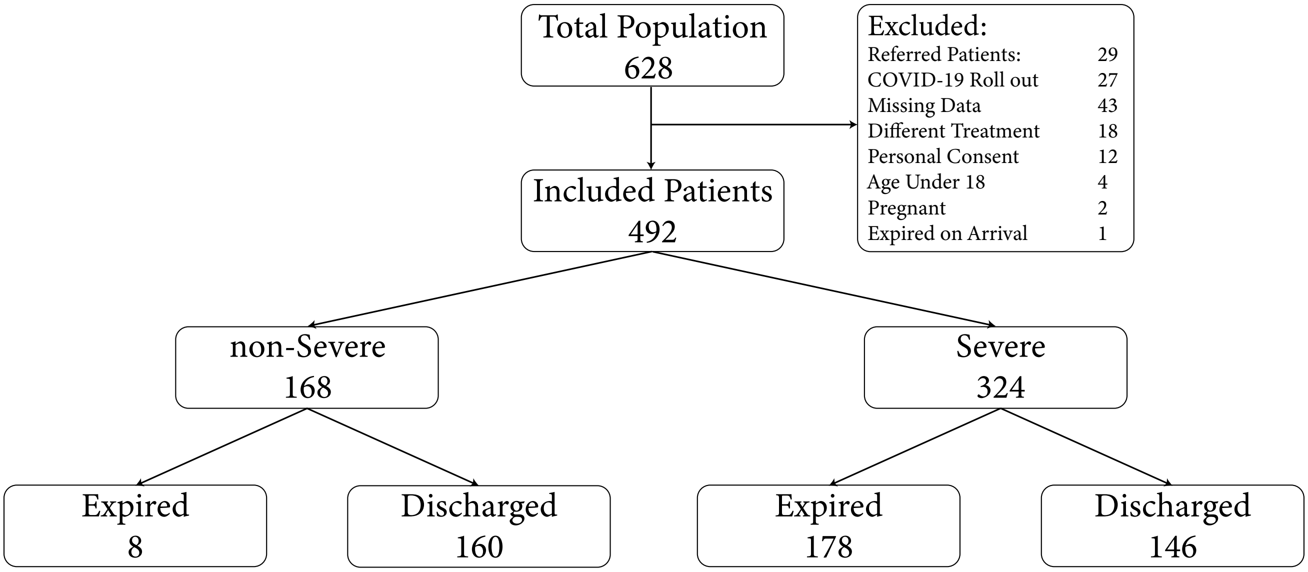

S1 Fig. Flowchart of the study population

SVM weights

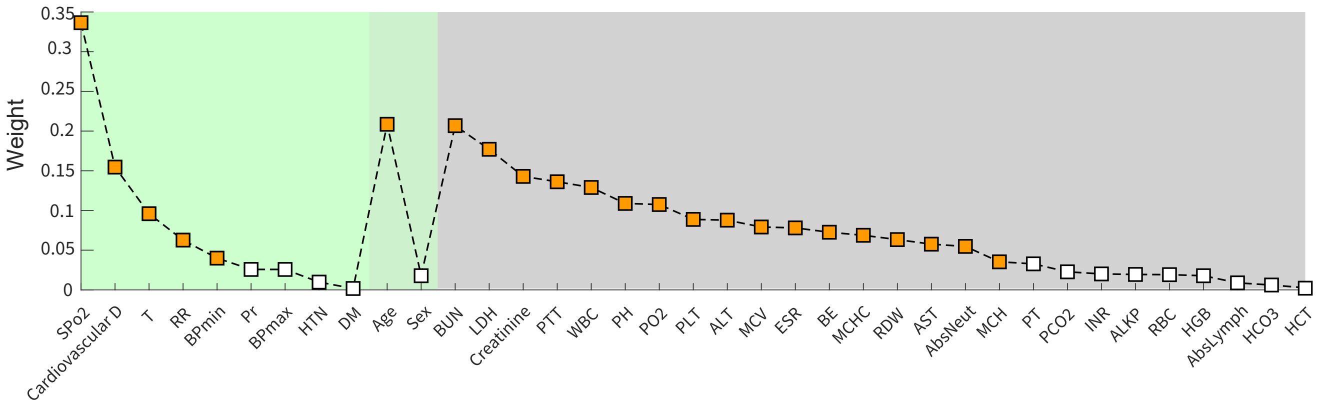

S2 Fig. Feature weights from the joint SVM model. Model weights are normalized to have a sum of one.

Feature analysis via generalized linear model with Lasso regularization

To evaluate the feature investigation results from the NCA analysis, generalized linear model with Lasso regularization (LassoGlm) [1] was utilized. Generalized linear models (Glm) are flexible expansions of ordinary linear regression to generalize their functionality to encompass situations where the outcome follows different probability distributions, such as a binomial distribution. Lasso is a regularization technique that utilizes a penalty term (λ) to constrains the value of estimated model coefficients, pushing them towards zero [2]. The LassoGlm technique is often used as a feature selection technique to identify features which are thought to be of significant importance for outcome prediction [3]. To implement this technique, the *lassoglm* command of MATLAB was utilized. All the data instances were used as inputs. Non-categorical features were zscore normalized and were passed into the model along with categorical features. The distribution of the outcome of LassoGlm was set to *binomial* and cross validation with 10 folds was added to the model (the *'CV'* input of the *lassoglm* was set to 10).

The supplementary S3A Fig displays the cross validated deviance of the LassoGlm fit. Deviance is a goodness of fit measure that ,for a binomial model, is defined as 2 times of the log likelihood of the left out data (for more details, see [4]). The lambda that corresponded to a deviance equal to one standard error (the green line in S3A Fig) away from the minimum expected deviance (the blue line) was utilized to extract the corresponding features with non-zero coefficients. A reduced Glm model with these features and their corresponding coefficients is able to provide predictions with errors that are roughly one standard deviation different from the minimum deviance of the full model, making these features suitable substitutes for the full feature spectrum. In other words, these features have high content of information for predicting the outcome. The results revealed that three non-invasive and four invasive features had significant non-zero coefficients (S3B Fig). Consistent with other results of the study, this analysis augments the fact that, indeed, non-invasive features could have significant roles in the prediction of the mortality prognosis.

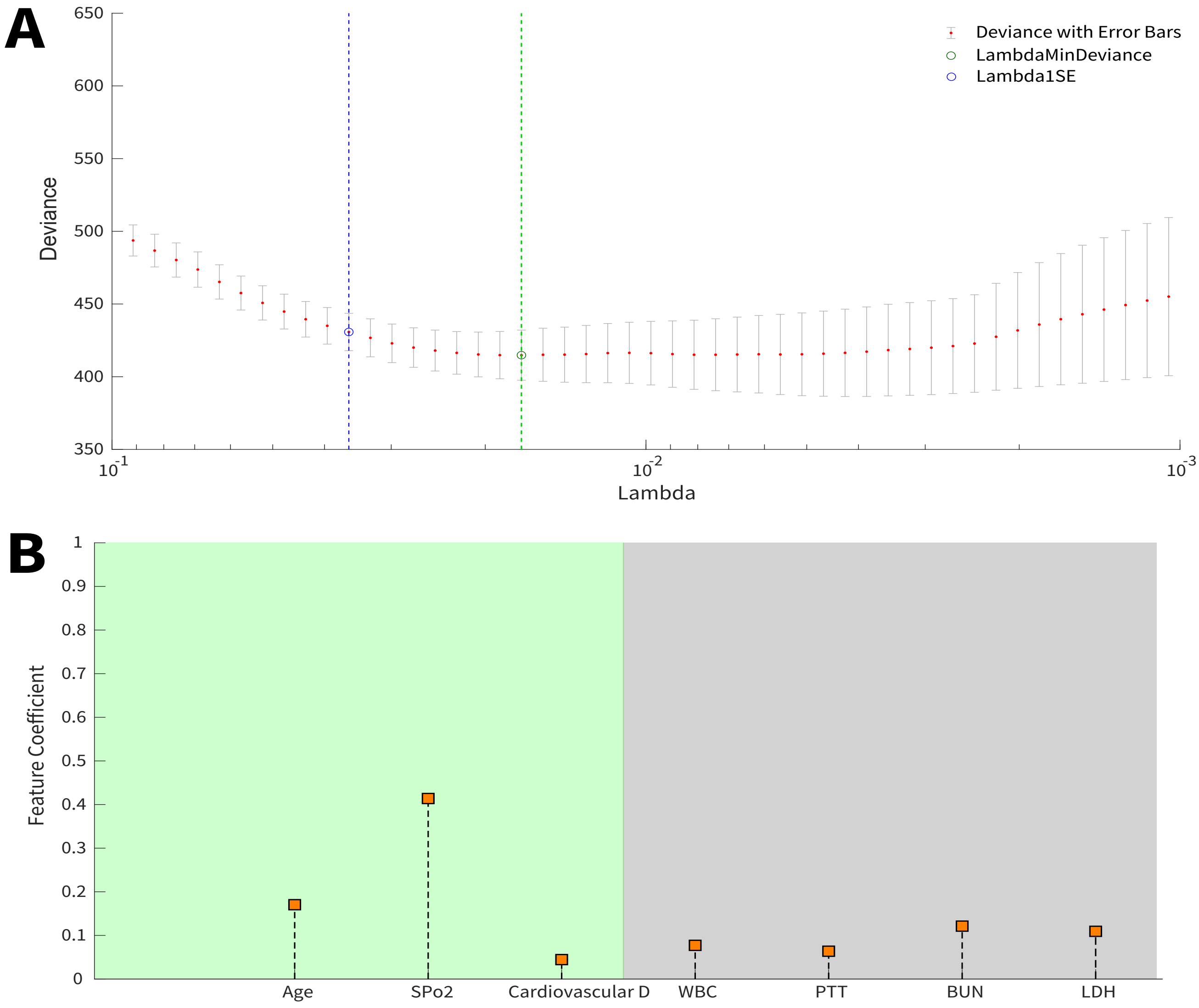

S3 Fig. Cross validated deviance and feature coefficients of the LassoGlm model. **(A)** 10 fold cross validated deviance of the LassoGlm fit. The Green circle and line displays the Lambda where the model has the minimum deviance. The blue circle and line show the Lambda where the model achieves deviance roughly 1 standard error away from the minimum deviance. **(B)** Features with non-zero coefficients obtained from the regularized Glm model. These features could be integrated in a reduced model that can provide predictions with deviances roughly one standard error away from the minimum deviance. Absolute values of feature coefficients with normalization to sum to one are reported here.

Ensemble model

To verify the results of our classification analysis, we performed additional modeling using a boosted ensemble of decision trees. At the beginning of the analysis the data was randomly shuffled and MATLAB function *cvpartition* was utilized to perform stratified separation of the data into training and test sets using a holdout ratio of 0.2. Model training was implemented on the training set via MATLAB function *fitcensemble*. Adaptive logistic boosting (*LogitBoost*) [5] was utilized as the boosting method for binary

classification. Number of ensemble trees, maximum number of node splits, and the learning rate were optimized using the *OptimizeHyperparameters* input of *fitcensemble*. The aforementioned input uses Bayesian optimization (*bayesopt*), as a default option, to find optimal values of hyperparameters in an optimization search range. Hyperparameter optimization ranges were tuned using *hyperparameters* command of the MATLAB which creates an optimizablevariable object; utilized ranges were as followed: number of ensemble trees [50 500], learning rate [0.1 0.8], and maximum number splits [1 10]. The *fitcensemble* command trained and optimized the hyperparameters on the training set across 60 evaluation iterations and 10 folds (*KFold* argument of *fitcensemble* was set to 10; the *repartition* argument was also set to *true* to decrease the effects of partitioning noise via repetition of kfold partitioning in each iteration). Feature weights were obtained from *PredictorImportanc* property of the model. The *kfoldPredict* MATLAB function was utilized to obtain class labels and scores for in-fold instances using a model trained on out-of-fold instances. Parameters for plotting the ROC curves were obtained from *perfcurve* function.

Supplementary Figure S4 shows that the non-invasive model was able to achieve performances on par with the joint model and slightly better than the invasive model. This analysis further affirms the results of our SVM analysis and the hypothesis that , as a rapid triage tool, it is possible to predict the mortality prognosis of COVID-19 patients using non-invasive features. Moreover, As displayed in S5 Fig, several non-invasive and invasive features had significant mortality prediction weights; noticeable among them were SPO_2, age, presence of cardiovascular disorders, Urea, LDH, and PTT. These results are consistent with results obtained from the LassoGlm, NCA, and SVM models. SPO_2 seems to particularly have significant mortality prediction weight in all of our models. Accordingly, physicians and health care staff should be alerted by patients whose in-room oxygen saturation (i.e., without oxygen support) are abnormal upon admission.

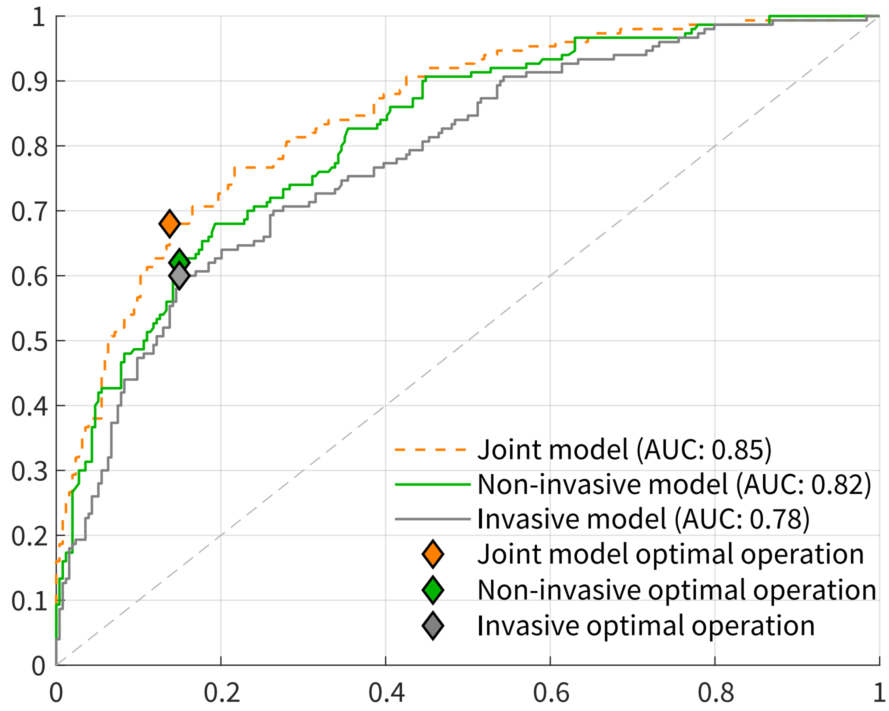

S4 Fig. Performance ROC curves of the joint, non-invasive, and invasive ensemble models

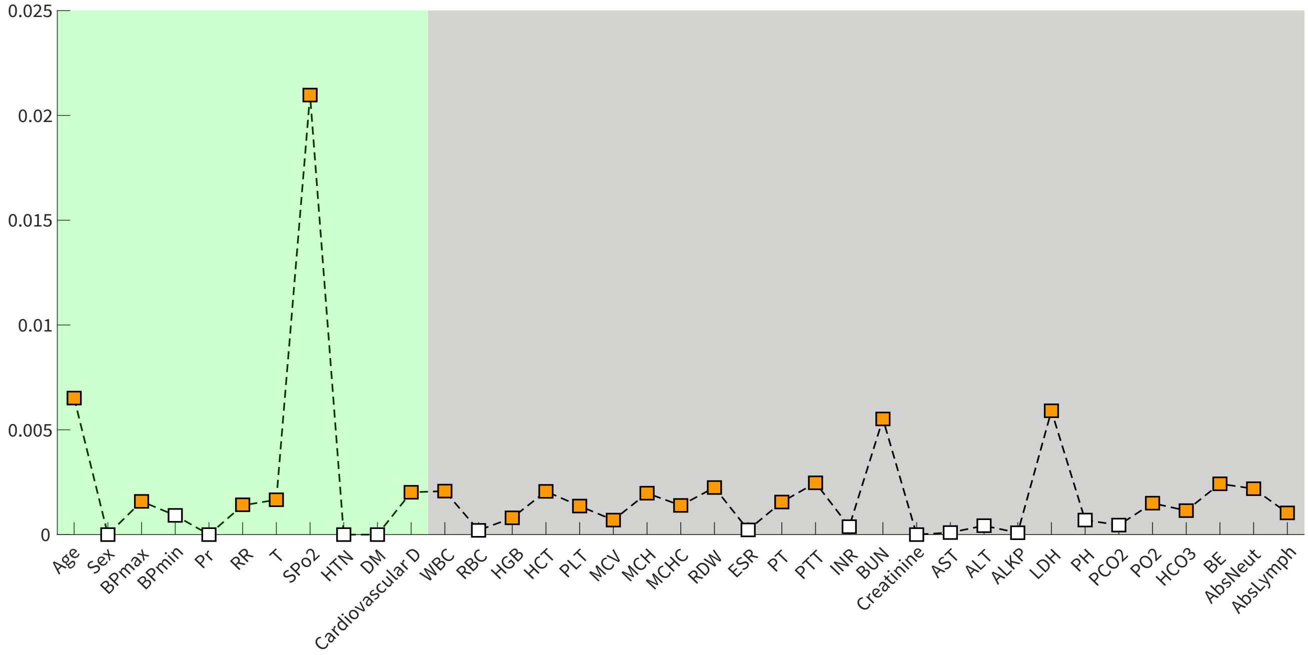

S5 Fig. Feature weights obtained from the cross-validated ensemble model. Feature weights were normalized to sum to 1.

References

1. Friedman, J., Hastie, T. & Tibshirani, R. Regularization paths for generalized linear models via coordinate descent. *Journal of statistical software* **33,** 1 (2010).

2. Tibshirani, R. Regression shrinkage and selection via the lasso. *Journal of the Royal Statistical Society: Series B (Methodological)* **58,** 267–288 (1996).

3. Fonti, V. & Belitser, E. Feature selection using lasso. *VU Amsterdam Research Paper in Business Analytics* **30,** 1–25 (2017).

4. Feng, C., Li, L. & Sadeghpour, A. A comparison of residual diagnosis tools for diagnosing regression models for count data. *BMC Medical Research Methodology* **20,** 1–21 (2020).

5. Friedman, J., Hastie, T., Tibshirani, R., *et al.* Additive logistic regression: a statistical view of boosting (with discussion and a rejoinder by the authors). *The annals of statistics* **28,** 337–407 (2000).
