# Supplementary material for: A machine learning based exploration of COVID-19 mortality risk
Source: PLoS One. 2021 Jul 2;16(7):e0252384. doi: 10.1371/journal.pone.0252384 (PMC8253432; doi:10.1371/journal.pone.0252384)
Supplement: Attachment — Submitted filename: Response to Reviewers.docx [file pone.0252384.s002.docx]

**Dear Dr. Usman Qamar,**

Academic Editor

PLOS ONE

Thank you for giving us the opportunity to resubmit our revised manuscript “*A machine learning based exploration of COVID-19 mortality risk”,* Ms. No: PONE-D-21-09671 to PLOS ONE. In this version of the manuscript, we have addressed all reviewer comments and made changes throughout the main manuscript text. We appreciate the time and effort that you and the reviewers have dedicated to providing your feedback on our manuscript. Below, we detail how we have responded to the reviewers’ comments.

Sincerely,

Ehsan Kamrani, Ph.D

Reza Lashgari, Ph.D

-----------------------------------------------------------------------------------

**Reviewer Points:**

-  Authors are to consider these models for developing countries?

**Answer:** Yes. The aim of this study is to pave the way for the implementation of computer-assisted triage in Iranian hospitals. As a developing country, Iran faced significant waves of patients that severely strained the healthcare system, causing a shortage of medical personnel and equipment. This study was conducted on Iranian patients in Masih Daneshvari Hospital, and the authors hope that by providing evidence for the performance of automated triage models in the Iranian population, healthcare systems of Iran and other developing countries be encouraged to support research, designation, and implementation of these models in medial environments to assist medical triage and decision making.

**Editorial comments:**

1. Please ensure that your manuscript meets PLOS ONE's style requirements, including those for file naming.

**Answer**: The manuscript was revised to meet the style requirements of the journal.

2. Please review your reference list to ensure that it is complete and correct. If you have cited papers that have been retracted, please include the rationale for doing so in the manuscript text, or remove these references and replace them with relevant current references. Any changes to the reference list should be mentioned in the rebuttal letter that accompanies your revised manuscript. If you need to cite a retracted article, indicate the article’s retracted status in the References list and also include a citation and full reference for the retraction notice.

**Answer**: Reference list was reviewed to check for quality. Several references were updated to be more complete.

3. Please amend your Ethics Statement and Methods section to state the type of informed consent provided (e.g., written, verbal, etc).

Answer: The ethics statement was amended to address this issue.

4.We note that you have indicated that data from this study are available upon request. PLOS only allows data to be available upon request if there are legal or ethical restrictions on sharing data publicly.

**Answer**: After consulting with the hospital’s committee of medical research, permission was granted for sharing of patient anonymized data. The url for the dataset is included in the cover letter.

5. Please include captions for your Supporting Information files at the end of your manuscript, and update any in-text citations to match accordingly

**Answer**: Captions for supporting information files were added to the end of manuscript and text was updated to match citations.
